# Exploring the prevalence and impact of hip and knee pain in pulmonary rehabilitation: a propensity-matched cohort study

**DOI:** 10.1186/s12931-022-02049-y

**Published:** 2022-06-03

**Authors:** Samuel Briggs-Price, Enya Daynes, Emma Chaplin, Sarah Ward, Linzy Houchen-Wolloff, Sally J. Singh

**Affiliations:** 1 Centre for Exercise and Rehabilitation Science, NIHR Leicester Biomedical Research Centre - Respiratory, Glenfield Hospital, Groby Road, Leicester, LE3 9QP UK; 2 Department of Pulmonary Rehabilitation, University Hospitals of Leicester, Glenfield Hospital, Groby Road, Leicester, LE3 9QP UK; 3 Department of Respiratory Sciences, University of Leicester, Leicester, UK

**Keywords:** Pulmonary rehabilitation, Pain, Hip, Knee, Musculoskeletal

## Abstract

**Background:**

Musculoskeletal pain is more common in individuals with chronic respiratory diseases than the aged-matched general population. This investigation aimed to understand the prevalence and impact of hip and knee pain on pulmonary rehabilitation outcomes and completion rates.

**Methods:**

Participants who experienced hip/knee pain in the 4 weeks prior to pulmonary rehabilitation completed an Oxford Hip and/or Knee Score alongside a routine pulmonary rehabilitation assessment. Participants engaged in a twice-weekly, 6-week outpatient pulmonary rehabilitation programme. A 1:1 propensity score match for age, sex, BMI, sessions attended and MRC score was completed prior to group comparison for a pulmonary rehabilitation cohort without hip/knee pain.

**Results:**

6.5% (n = 97) of pulmonary rehabilitation participants reported pain: hip (n = 27), knee (n = 40) or hip and knee pain (n = 30). 75 participants with hip/knee pain provided sufficient data for pre pulmonary rehabilitation matching and were propensity matched with a pulmonary rehabilitation group without hip/knee pain. The average Oxford Score across all reported joints was 28.7 (8.5) indicating moderate/severe pain at baseline. Statistically significant improvements were made in Oxford Scores for the left hip, left knee and right knee (P < 0.01) but not the right hip following pulmonary rehabilitation. There was no statistically significant difference between groups for improvements in quadriceps strength, walking tests or depression scores, both groups achieved within group significance. There were no significant differences in pulmonary rehabilitation completion rates between groups.

**Conclusions:**

Prevalence of hip/knee pain in individuals presenting to pulmonary rehabilitation is 6.5%. Pain improved in the majority of joints following pulmonary rehabilitation and pain did not impact the effectiveness or completion of the programme.

*Trial Registration:* This trial was an evaluation of a clinical service and has not been registered in a public domain.

**Supplementary Information:**

The online version contains supplementary material available at 10.1186/s12931-022-02049-y.

## Background

Chronic respiratory diseases (CRD) are common and associated with increased morbidity and premature mortality [1]. Pulmonary rehabilitation (PR) is the cornerstone for CRD management and has a strong evidence base [2]. PR comprises of multidisciplinary led, individually prescribed exercise and supported self-management education that aims to increase a patient’s exercise capacity, improve quality of life and promote self-management [3]. The intervention has a favourable impact on acute and emergency admissions to drive down health care utilisation [4].

Despite the established benefits of PR in CRD, other (non-respiratory) factors can impact on an individual’s ability to engage in PR. Musculoskeletal (MSK) pain is more prevalent and severe in individuals with CRD’s compared to the general population [5, 6]. MSK pathology such as osteoarthritis is a leading cause of pain in individuals attending PR and is associated with poor self-reported health status [7, 8]*.* National guidelines advise that individuals with severe arthritis should not be referred to PR [9], although there is no evidence to suggest why this patient group cannot engage with or benefit from PR. Both individuals with CRD’s and their healthcare professionals propose hip and knee pain as a barrier affecting an individual’s ability to complete PR sessions [10, 11]. It remains unclear whether individuals with hip and knee pain will tolerate PR to achieve its benefits without aggravating hip or knee symptoms.

Specific group based exercise interventions for participants with hip and knee osteoarthritis are effective for improving pain, exercise capacity and self-reported function [12, 13]. These programmes share many common features with PR in both format and content and do not exclude individuals with CRD. However, unlike current osteoarthritis programmes, PR does not offer formal education on MSK pain, pathology or management. Exercise prescription is symptom guided in both programmes but may potentially differ, PR frequently uses perceived shortness of breath (SOB) scales and MSK programmes use pain irritability to calibrate the exercise prescription. In addition, PR primarily prescribes exercises to improve aerobic fitness whereas osteoarthritis programmes primarily target strength and neuromuscular deficits. Ideally, individuals with CRD and hip or knee pain require an intervention which addresses the clinical features of both conditions.

There has been several reports focusing on the impact of comorbidities on the outcome of PR but there is a lack of data specifically looking at pain as a symptom [14, 15]. Previous prospective observational studies and retrospective reviews have investigated the impact of pain at various body sites on PR [16, 17], finding pain to have negligible influence on PR outcomes or completion. In contrast, Butler [15] found the presence of MSK conditions significantly impacted on the ability to secure improvements in exercise capacity in individuals with interstitial lung disease (ILD). However, these investigations did not identify the influence of pain severity or specific pain locations on PR outcomes. This presents a significant challenge when interpreting the impact of MSK pain on PR. For example, the location of pain may influence the impact on PR outcomes. PR programmes typically include pre and post walking tests and it is unlikely that upper body MSK pain would influence walking test outcomes. Therefore previous studies [15, 16] analysing the impact of lower and upper body pain together may misrepresent the influence of MSK pain on PR physical outcomes.

Our primary aims were to identify the prevalence of lower limb (hip and/or knee) pain in those presenting to PR and to understand the impact of hip and knee pain on PR outcomes and completion, compared to a matched population without lower-limb pain. A secondary aim was to describe the change in self-reported hip and knee pain and function following PR.

## Methods

### Participants

We conducted a retrospective analysis of data collected from individuals with CRD enrolled in a PR programme at University Hospitals of Leicester NHS Trust between June 2016 and August 2019. Individuals with CRD were referred to PR by respiratory consultants, general practitioners (GP), respiratory specialist nurses, allied health professionals or from staff on respiratory wards.

Participants who enrolled onto PR attended an individualised initial assessment. Standard outcome measures were completed including the incremental shuttle walking test (ISWT)[18] and endurance shuttle walk test (ESWT)[19]. The reason for terminating each walking test was recorded by the clinician conducting the test (e.g. dyspnoea, pain, fatigue). Quadriceps maximal voluntary contraction (QMVC) was measured using a chair based dynamometer [20]. A range of symptom based scales and questionnaires were completed including the Medical Research Council (MRC) Dyspnoea Scale [21] and hospital anxiety and depression score (HADS) [22]. All participants were asked at initial assessment if they had experienced hip or knee pain over the past 4 weeks. Participants who reported pain were requested to complete the Oxford Knee Score and/or Oxford Hip Score [23, 24] questionnaires for the affected joints pre and post PR. Each score comprises of 12 equally weighted questions addressing the patient’s perceived pain and functional activity answered on a Likert scale with values form 0 to 4, with a reference range of the last 4 weeks. The total score ranges from 0 to 48 and is categorised in the following thresholds: 0–19 severe, 20–29 moderate-severe, 30–39 mild-moderate and 40–48 satisfactory pain.

Participants engaged in a twice-weekly, 6-week outpatient PR programme, comprising of supervised exercise and group-based education. The PR programme adhered to current British Thoracic Society guidelines [1]. This included aerobic exercise of individually prescribed walking and static cycling. The resistance component consisted of upper and lower limb strength training using dumbbells with the load being individually prescribed by the therapist. Participants were also advised to complete an unsupervised home-exercise programme which reflected the supervised hospital programme of walking and resistance training. All outcome measures were repeated upon completion of the programme. Completion was defined as attending a discharge assessment. The number of sessions each individual attended was also recorded.

### Data analysis

Data was analysed using SPSS version 26.0 (IBM, New York, USA). Demographic data and reasons for stopping the walking tests was analysed for the entire sample with hip and knee pain. Distribution of data was assessed for normality and was reported as means with standard deviation.

Propensity score matching was completed between participants with hip and knee pain compared to participants with no hip and knee pain. A 1:1 nearest neighbour propensity score-match was completed using age, body mass index (BMI), gender, sessions completed and MRC score. When analysing completion rates, the number of sessions completed was not included in matching.

Independent t-tests were used to compare outcomes between groups. Paired t-test was used to compare pre and post rehabilitation outcomes within groups. P value set at p < 0.05.

Participants were included for analysis if they had given their written consent for their data from the PR assessment to be recorded and evaluated for audit purposes.

## Results

Over 26 months 1492 participants were assessed and started PR, 6.5% (n = 97) of participants reported hip or knee pain at baseline assessment and completed the Oxford Hip and/or Knee Score (Hip 27.8%, Knee 41.3%, Hip and Knee pain 30.9%).

Demographics and distribution of primary respiratory diagnosis are displayed in Tables 1 and 2. There was no significant difference between groups described by pain location in pre-programme percentage predicted forced expiratory volume in one second (%FEV_1_).

**Table 1:** Group demographics prior to matching

Demographics Mean (SD)	PR comparison (n = 1395)		Hip and knee pain (n = 97)	
Age (year)	68.2 (10.54) (n = 1394)		69.3 (8.91) (n = 97)	
Male (%)	52.0% (n = 762)		55.7% (n = 54)	
%FEV_1_	58.61 (24.38) (n = 688)		75.95 (27.64) (n = 40)	
BMI (kg/m^2^)	28.23 (7.83) (n = 631)		32.32 (8.69) (n = 82)	
PR Sessions Attended	7.37 (5.28) (n = 1299)		10.22 (3.07) (n = 89)	
MRC Grade n (%)	1	14 (1.1%)	1	1 (1%)
	2	286 (22.4%)	2	20 (20.8%)
	3	474 (37.2%)	3	42 (43.8%)
	4	387 (27.7%)	4	22 (22.9%)
	5	113 (8.9%)	5	11 (11.5%)

The values are expressed as means (SD) (n = number of participants). FEV_1_ = Forced Expiratory Volume in 1 Second *BMI*  body mass index, *PR* pulmonary rehabilitation, *MRC* Medical Research Council Dyspnoea Scale

**Table 2:** Primary respiratory diagnosis prior to matching

Primary respiratory diagnosis (%)	PR comparison (n = 1395)	Hip and knee pain (n = 97)
COPD	68.1% (n = 948)	65.9% (n = 64)
Bronchiectasis	7.3% (n = 102)	7.2% (n = 7)
Asthma	7.8% (n = 108)	4.1% (n = 4)
ILD	11.0% (n = 153)	14.4% (n = 14)
Other restrictive	0.2% (n = 3)	0.0% (n = 0)
Other	5.6% (n = 78)	8.2% (n = 8)

The values are expressed as percentages (n = Number of participants). *COPD* chronic obstructive pulmonary disease, *ILD* interstitial lung disease, *PR*  pulmonary rehabilitation, *Other* undiagnosed breathlessness, obstructive sleep apnoea, multi-dimensional breathlessness

Collectively for all joint pain, severity was reported as 12% severe, 38% moderate-severe, 37% mild-moderate and 13% satisfactory. The average Oxford Hip and Knee Score reported for the 97 participants across hip and knee joints was 28.7 (8.5), indicating moderate/severe joint pain.

Of the 97 participants reporting hip or knee pain, 75 provided sufficient data for propensity matching. The 75 were matched with 75 contemporary participants who attended PR without hip or knee pain. Distributions of propensity scores are provided in Additional file 1.

Within the hip and knee pain group, 90.6% of participants reported at least one additional comorbidity, with an average of 4.1 comorbidities in total. Within the PR comparison group, 78.7% of participants reported at least one additional comorbidity, with an average of 3.7 comorbidities in total. There was no significant difference in the mean number of comorbidities between groups (P = 0.56).

**Fig. 1:**
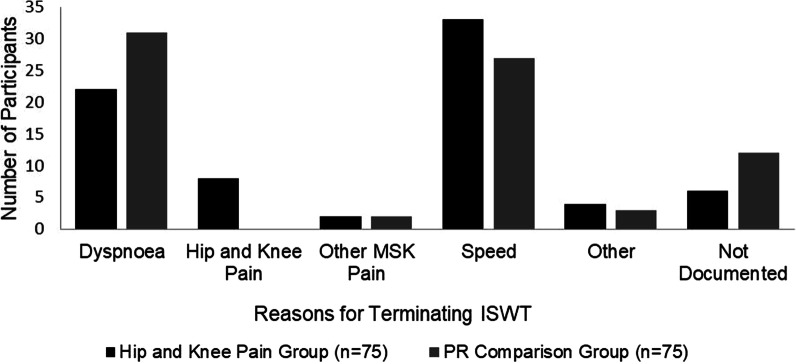
Reasons for terminating pre-PR ISWT

Both pain and no pain groups made significant improvements in the ISWT, ESWT, QMVC, MRC and HADS depression subscale (Table 3). No significant difference was found between groups for any outcome except the HADS Anxiety subscale which improved significantly only in the group without hip or knee pain (Table 3). Reasons for terminating the pre-PR ISWT for matched groups have been displayed in Fig. 1.

**Table 3:** Group outcomes pre and post PR with between group comparisons

	HKP Mean (SD)	HKP mean difference (95%CI)	PR control	PR control mean difference (95%CI)	Between group differences
ISWT pre (metres)	241.1 (141.2)	66.8*** (50.6, 83.0)	206.6 (120.9)	81.8*** (61.7, 101.9)	15.0
ISWT post	307.9 (141.3)		288.4 (140.9)		
ESWT pre (secs)	205.8 (121.8)	412.9*** (319.4, 506.4)	212.8 (106.0)	353.6*** (263.2, 444.0)	− 59.3
ESWT post	618.8 (377.1)		566.4 (367.9)		
QMVC pre (kg)	21.1 (11.9)	4.4** (− 7.1, 1.6)	22.6 (10.0)	4.5*** (2.4, 6.5)	0.1
QMVC post	25.5 (12.7)		27.1(11.0)		
MRC pre	3.2 (0.9)	0.7*** (0.4, 0.9)	3.1 (0.9)	0.3** (0.1, 0.6)	0.4
MRC post	2.5 (0.8)		2.8 (1.0)		
HADS A pre	6.9 (4.1)	0.2 (0.6, 1.1)	7.0 (3.9)	1.4** (0.6, 2.3)	1.2*
HADS A post	6.7 (4.0)		5.6 (3.7)		
HADS D pre	6.1 (3.5)	1.1* (0.1, 2.1)	6.3 (3.7)	1.6** (0.9, 2.4)	− 0.5
HADS D post	5.0 (3.7)		4.7 (3.6)		

Group values are expressed as means (SD). *P < 0.05, **P < 0.01 ***P < 0.001. *MD*  mean difference between groups (95%Confidence Interval). *HKP*  participants with hip and knee pain, *PR*  pulmonary rehabilitation, *ISWT*  incremental shuttle walk test, *ESWT*  endurance shuttle walk test, *QMVC*  quadriceps maximal voluntary contraction, *MRC*  Medical Research Council Dyspnoea Scale, *HADS* *A*  hospital anxiety and depression scale anxiety sub score, *HADS D* hospital anxiety and depression scale depression sub score

**Fig. 2:**
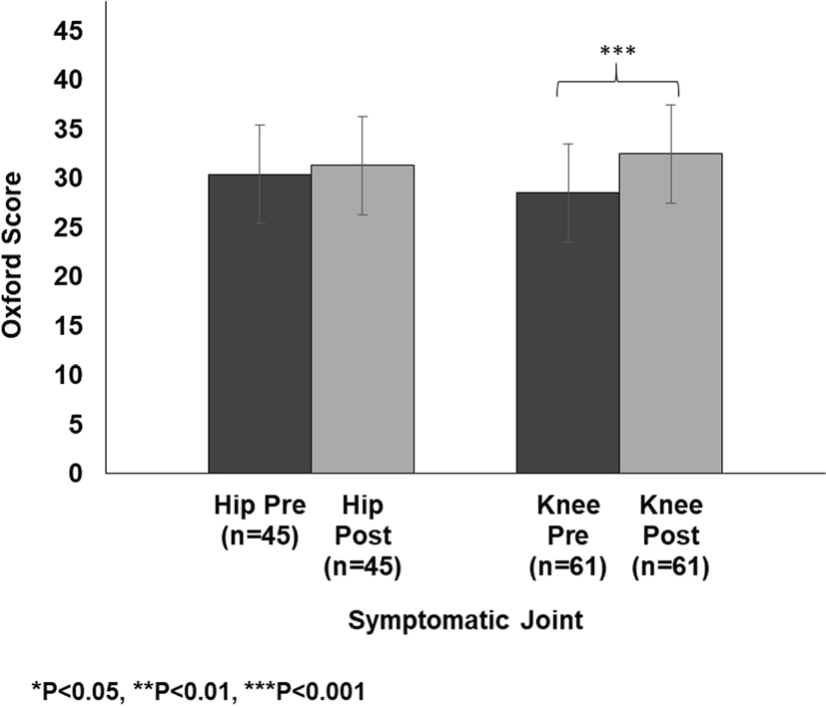
Oxford Scores for matched participant’s pre and post PR

For the matched participants significant improvements in Oxford Scores were seen for the left hip, right knee and left knee (P < 0.01). No significant improvement or deterioration was seen in the right hip. There was no significant difference in baseline Oxford Scores between joint sites. Pre and post PR Oxford Score changes for the hip and knee are presented in Fig. 2. Severe pain sites made the largest improvement (6.92), followed by moderate (3.35) and mild sites (2.25). Satisfactory pain sites were the only sites to have a decrease in Oxford Score (-5.7).

**Fig. 3:**
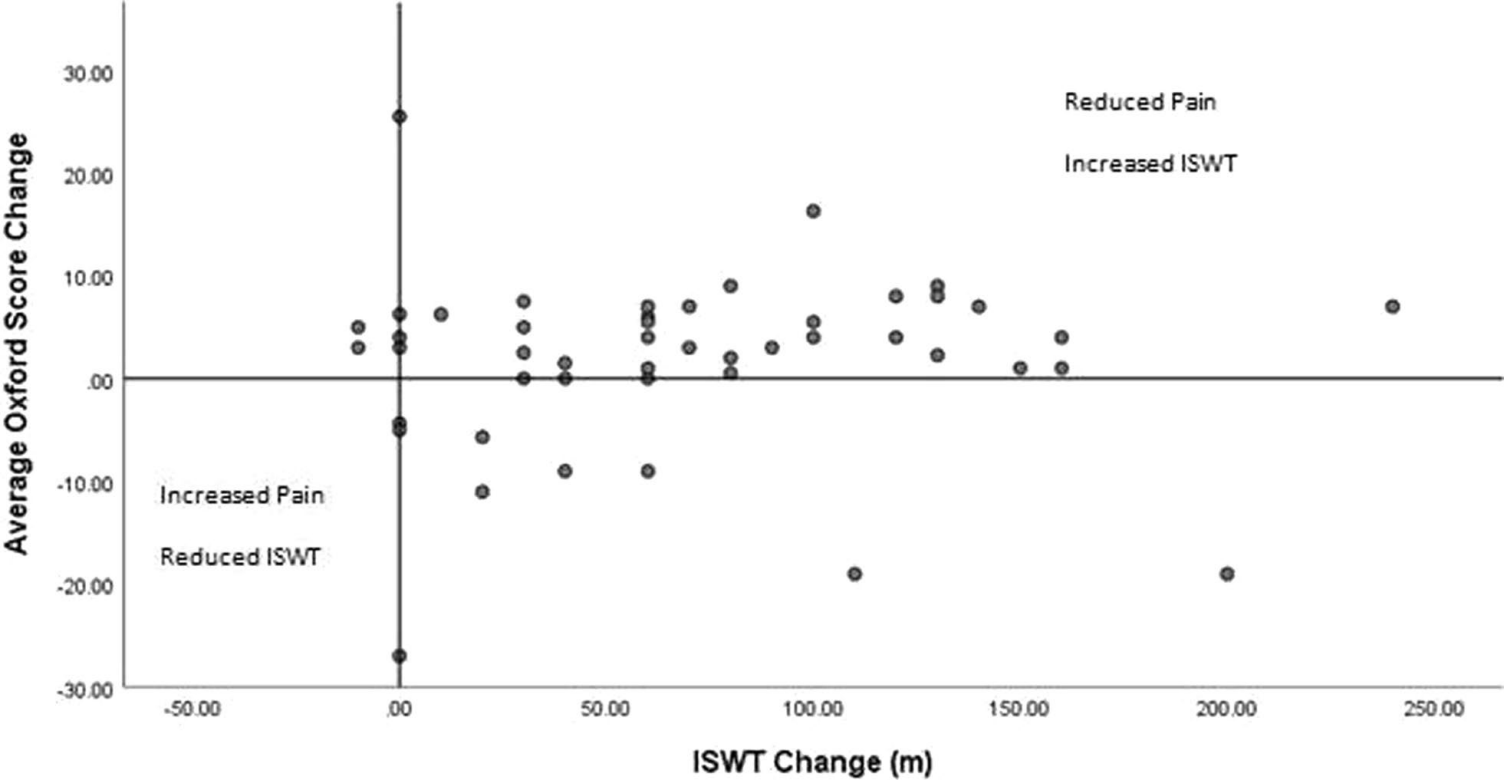
Average Oxford Score change and ISWT change

**Fig. 4:**
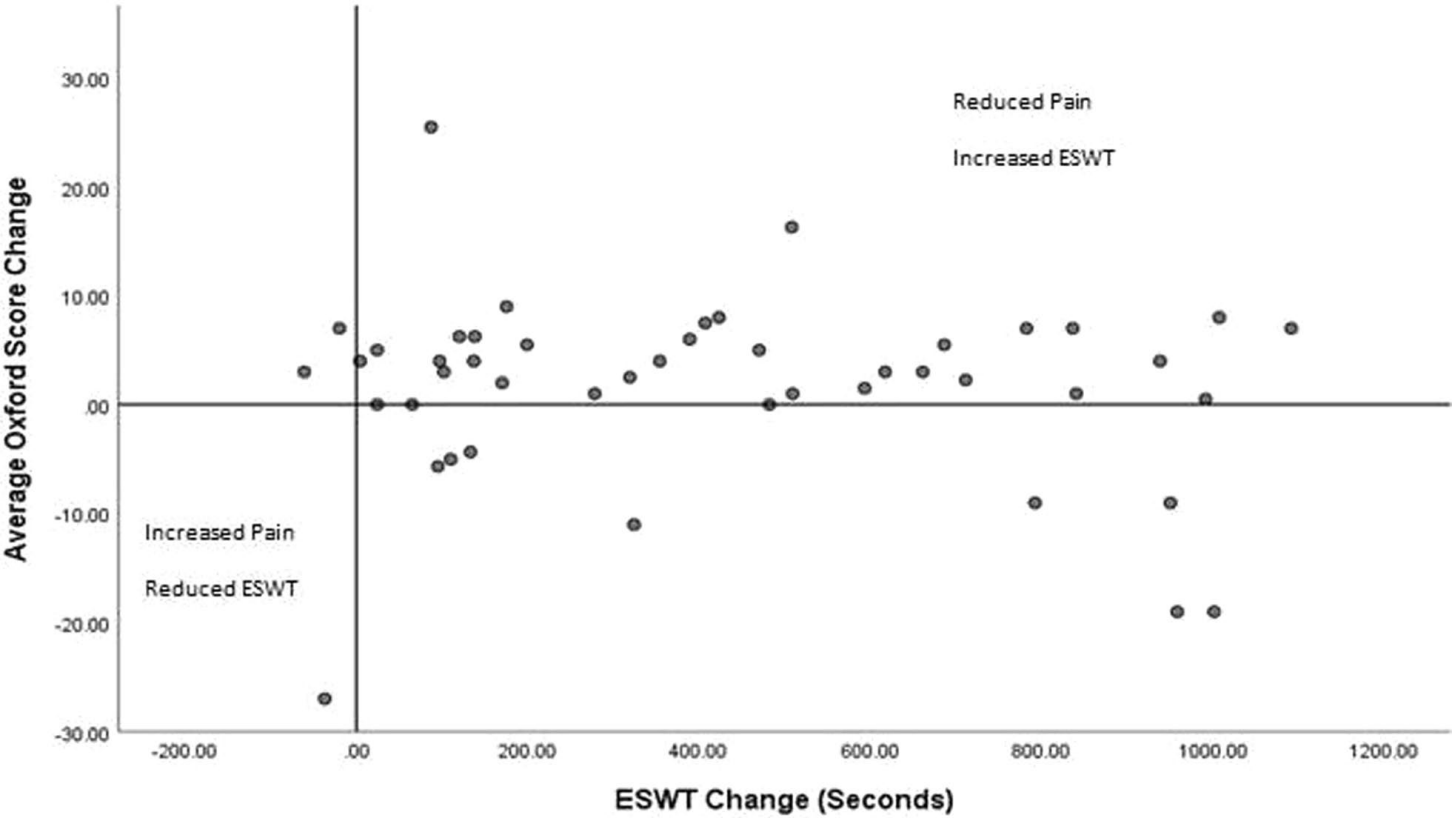
Average Oxford Score change and ESWT change

The majority of participants with hip and knee pain (71.8%) improved in both walking performance and pain scores. No participants experienced a decrease in ISWT and Oxford Score (Fig. 3). Only one participant experienced a reduction in ESWT distance and Oxford Score (Fig. 4).

Matching was repeated between groups without sessions completed included. The hip and knee pain group completed a mean (SD) of 10 (3) sessions in comparison to 11 (3) in the PR group without hip/knee pain. There were 20 non-completers in the hip and knee pain group and 19 in the group without hip/knee pain (p = 0.85). The primary reason for non-completion in each group was the participant not contacting the PR department or a reason not being documented (Hip and knee pain n = 12, PR comparison n = 11). Following this, the reasons for non-completion in the hip and knee pain group were hip and knee pain (n = 3), social (n = 3) and other comorbidities (n = 2). In the PR comparison group the reasons for non-completion were other comorbidities (n = 6) and social (n = 2).

## Discussion

This data describes the impact of lower limb pain on the outcome of PR compared to a contemporaneous group with no lower limb pain. Importantly, it identifies that the presence of lower limb pain does not compromise the outcome of PR, and the musculoskeletal pain was not aggravated by the intervention. In fact it appears that PR has a beneficial impact on lower limb pain.

Reported pain scores for both the hip and knee significantly reduced at the majority of pain sites following PR. Importantly hip and knee pain did not influence the outcomes of ISWT, ESWT, QMVC, MRC and HADS depression subscale following PR. Furthermore, there was no significant difference in completion rates between groups confirming that PR does not aggravate hip and knee pain and it does not appear to be a cause for excess dropout. Both groups with and without hip and knee pain exceeded the minimum important difference for the ISWT (35 m) and ESWT (174 s) [25, 26].

The prevalence of hip and knee pain was lower than anticipated. Hip and knee pain during this period represents active pain sites and therefore prevalence is lower than studies reporting a recorded history of MSK comorbidities in PR [15, 27, 28]. The low prevalence seen may also reflect a referral bias as previous research has identified that individuals with a painful condition were less likely to be referred to PR by primary care teams [29]. The decision by referrers to defer PR whilst individuals with CRD are experiencing lower limb pain may be informed by current guidelines. National guidelines advise that individuals with severe orthopaedic conditions are not appropriate for PR referral [9]. However, participants within this study included all self-reported severity levels and increased severity was not predictive of pain improvement.

If individuals with CRD and hip or knee pain are referred they may choose to not attend their PR assessment due to fear of aggravating their symptoms. Qualitative investigations have identified patient anxieties regarding aggravating symptoms and worsening pathology through PR [30]. In this investigation the HADS Anxiety sub score in the hip and knee pain group was unchanged by PR, unlike the group without hip and knee pain. However, both groups had low anxiety scores pre PR.

Importantly, PR did not aggravate self-reported hip or knee pain and function in individuals with CRD. The PR programme shares many components with effective lower limb rehabilitation regimes used in the conservative management of hip and knee conditions [12, 13]. Both of these established programmes achieve clinically meaningful improvements in hip and knee pain and function and health related quality of life. Following PR participants met the minimum important difference for the Oxford Knee Score but not the Oxford Hip Score [31]. Unlike MSK specific rehabilitation programmes, PR does not provide structured education sessions on MSK disease. Within PR, MSK disease education is informal and individualised to patient questions. Individuals with CRD feel a physiotherapist is the most qualified clinician to deliver a pain intervention in PR [11]. Although the advice provided by PR clinicians and whether this patient group would benefit from formal MSK disease education sessions is unknown.

### Limitations

We were unable to influence referral patterns and therefore have no data on the number of people who declined an offer of referral to rehabilitation because of hip or knee pain. The duration of hip and knee pain prior to PR was not gathered; therefore it is possible that participants who had experienced mild pain of short duration were included in analysis. Five participants reported an Oxford Score indicating no impairment despite reporting pain at PR initial assessment. This may provide an insight into why the ‘satisfactory’ severity group experienced a decline in Oxford Score.

The time frame of the Oxford Hip and Knee Scores has a reference point of 4 weeks which of course covers a significant proportion of the rehabilitation programme. The use of analgesia is not routinely recorded during pre and post PR assessments; it is unknown whether this influenced PR outcomes or Oxford Hip and Knee Scores.

Further research is warranted to investigate the impact of MSK comorbidities on referral to PR. Understanding the barriers for referrers and individuals with MSK pain to attending PR would be useful to tailor the offer and delivery of rehabilitation to this group. In addition, longer term follow up would provide an insight into how hip and knee pain interacts with post PR self-management strategies.

## Conclusion

Individuals with hip and knee pain did not have inferior outcomes in physical testing or decreased completion rates after PR, compared to those without hip/knee pain. Significant improvements were seen in the majority of pain sites following PR regardless of increased severity. This data challenges the national guidance and should favourably influence referral to PR for those with CRD and hip and knee pain and should inform conversations about PR between patients with hip and knee pain and referrers.

## Supplementary Information

**Additional file 1.** Distribution of propensity scores.

## Data Availability

The datasets used and/or analysed during the current study are available from the corresponding author on reasonable request.

## Abbreviations

CRD: Chronic respiratory diseases
PR: Pulmonary rehabilitation
MSK: Musculoskeletal
SOB: Shortness of breath
ILD: Iterstitial lung disease
GP: General practitioner
ISWT: Incremental shuttle walk test
ESWT: Endurance shuttle walk test
QMVC: Quadriceps maximal voluntary contraction
MRC: Medical Research Council
HADS: Hospital anxiety and depression score
BMI: Body mass index
SD: Standard deviation
