# Supplementary figures and images for: Exploring the prevalence and impact of hip and knee pain in pulmonary rehabilitation: a propensity-matched cohort study
Source: Respir Res. 2022 Jun 3;23:146. doi: 10.1186/s12931-022-02049-y (PMC9164348; doi:10.1186/s12931-022-02049-y)

**Additional material** – **Distribution of propensity Scores**


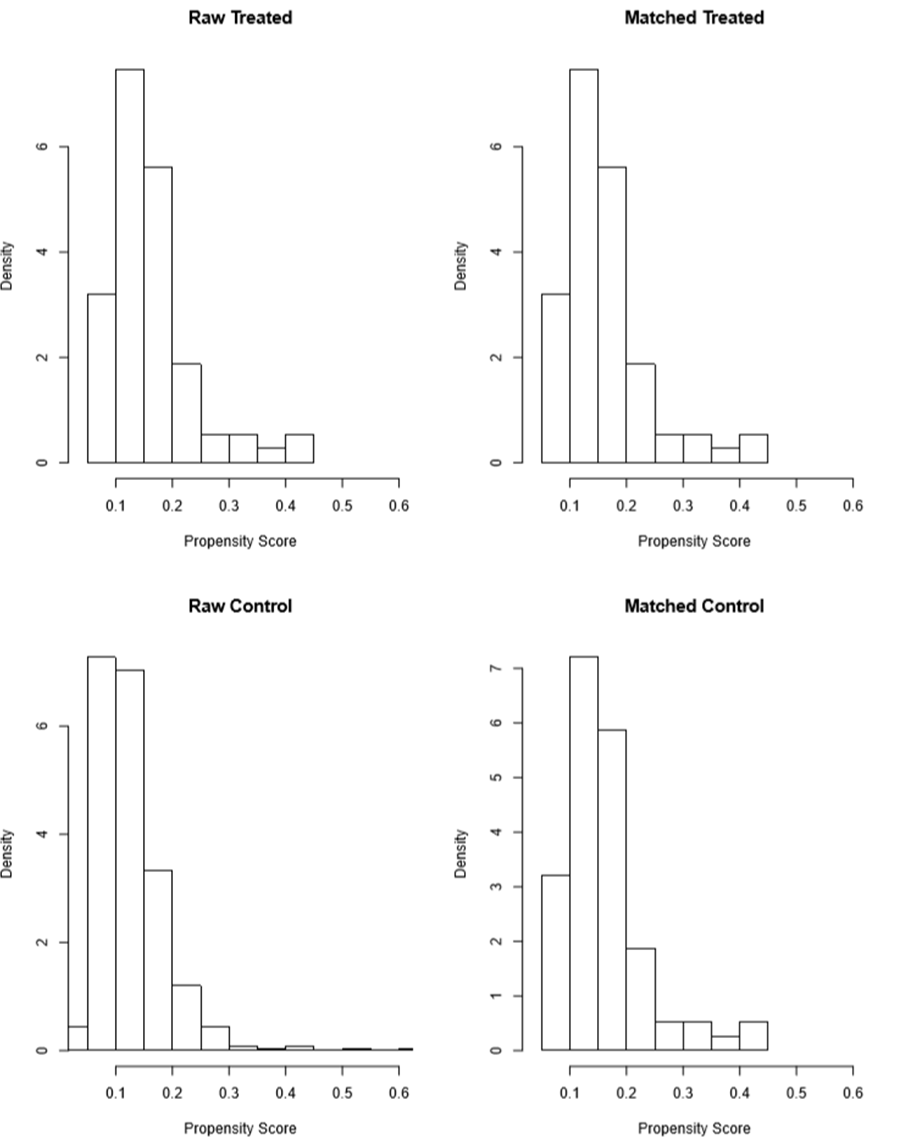


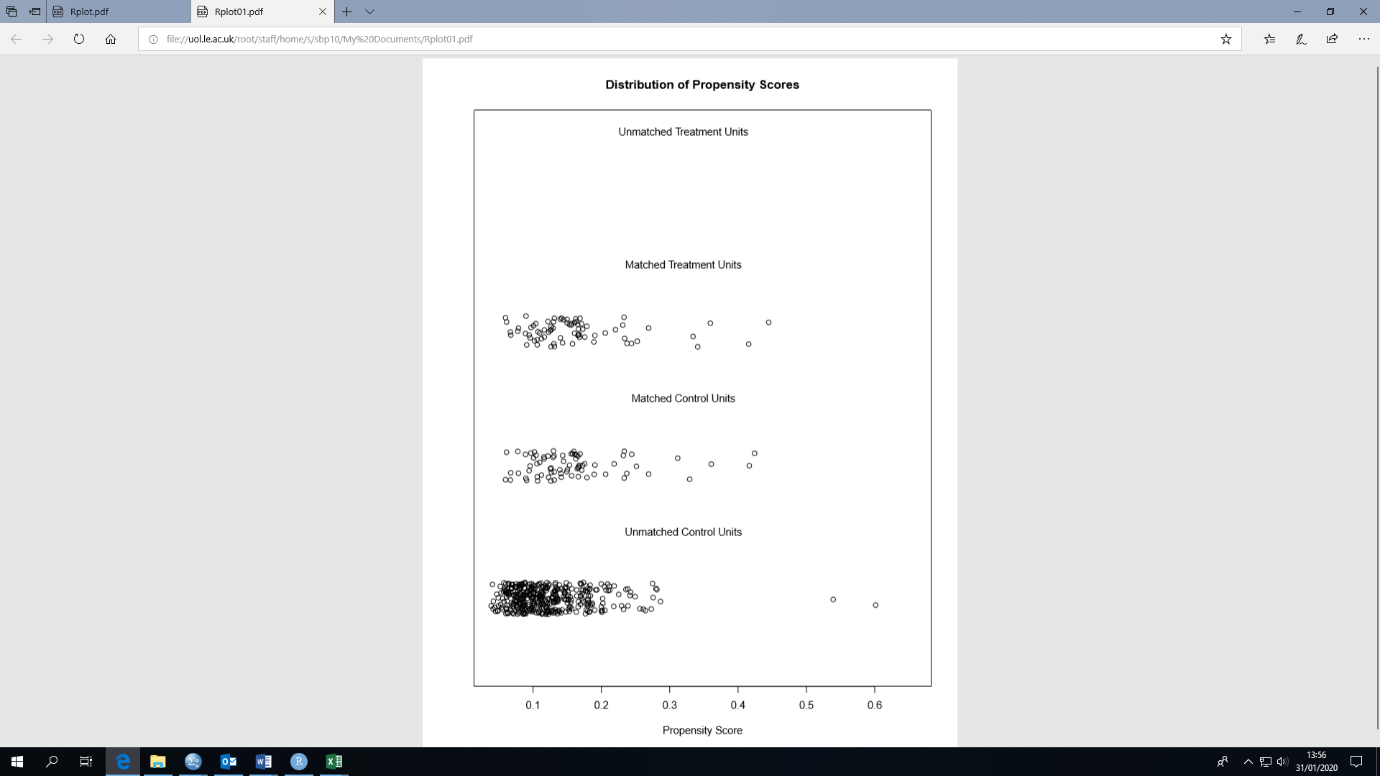

Supplement: Supplementary file 1 — Additional file 1. Distribution of propensity scores. [file 12931_2022_2049_MOESM1_ESM.docx]
